# Genome-Wide Analysis of Grain Yield Stability and Environmental Interactions in a Multiparental Soybean Population

**DOI:** 10.1534/g3.117.300300

**Published:** 2017-12-07

**Authors:** Alencar Xavier, Diego Jarquin, Reka Howard, Vishnu Ramasubramanian, James E. Specht, George L. Graef, William D. Beavis, Brian W. Diers, Qijian Song, Perry B. Cregan, Randall Nelson, Rouf Mian, J. Grover Shannon, Leah McHale, Dechun Wang, William Schapaugh, Aaron J. Lorenz, Shizhong Xu, William M. Muir, Katy M. Rainey

**Affiliations:** * Department of Agronomy, Purdue University, West Lafayette, Indiana 47907; † Department of Agronomy and Horticulture, University of Nebraska-Lincoln, Nebraska 68583; ‡ Department of Statistics, University of Nebraska-Lincoln, Nebraska 68583; § Department of Agronomy, Iowa State University, Ames, Iowa 50011; ** Department of Crop Sciences, University of Illinois, Urbana, Illinois 61801; †† United States Department of Agriculture (USDA)-Agricultural Research Service (ARS), Beltsville, Maryland 20705; ‡‡ USDA-ARS, Urbana, Illinois 61801; §§ USDA-ARS, Raleigh, North Carolina 27607; *** Department of Crop and Soil Sciences, North Carolina State University, Raleigh, North Carolina 27607; ††† Department of Plant Sciences, University of Missouri, Portageville, Missouri 63873; ‡‡‡ Department of Horticulture and Crop Sciences, Ohio State University, Columbus, Ohio 43210; §§§ Department of Plant Sciences, Michigan State University, East Lansing, Michigan 48824; **** Department of Agronomy, Kansas State University, Manhattan, Kansas 66506; †††† Department of Agronomy and Plant Genetics, University of Minnesota, Saint Paul, Minnesota 55108; ‡‡‡‡ Botany and Plant Sciences, University of California, Riverside, California 92521; §§§§ Department of Animal Sciences, Purdue University, West Lafayette, Indiana 47907

**Keywords:** Finlay-Wilkinson index, GGE, association mapping, meta-analysis, SoyNAM, Multiparent Advanced Generation Inter-Cross (MAGIC), multiparental populations, MPP

## Abstract

Genetic improvement toward optimized and stable agronomic performance of soybean genotypes is desirable for food security. Understanding how genotypes perform in different environmental conditions helps breeders develop sustainable cultivars adapted to target regions. Complex traits of importance are known to be controlled by a large number of genomic regions with small effects whose magnitude and direction are modulated by environmental factors. Knowledge of the constraints and undesirable effects resulting from genotype by environmental interactions is a key objective in improving selection procedures in soybean breeding programs. In this study, the genetic basis of soybean grain yield responsiveness to environmental factors was examined in a large soybean nested association population. For this, a genome-wide association to performance stability estimates generated from a Finlay-Wilkinson analysis and the inclusion of the interaction between marker genotypes and environmental factors was implemented. Genomic footprints were investigated by analysis and meta-analysis using a recently published multiparent model. Results indicated that specific soybean genomic regions were associated with stability, and that multiplicative interactions were present between environments and genetic background. Seven genomic regions in six chromosomes were identified as being associated with genotype-by-environment interactions. This study provides insight into genomic assisted breeding aimed at achieving a more stable agronomic performance of soybean, and documented opportunities to exploit genomic regions that were specifically associated with interactions involving environments and subpopulations.

One of the objectives of plant breeders is to develop cultivars that are high yielding across extensive range of environmental conditions. However, the presence of genotype-by-environment interactions (GEI) (Crossa 1990; Kang 1997; Zobel *et al.* 1988) might complicate this labor. For example, the GEI of a cross-over type causes changes in ranking performance across environments, complicating the breeder’s task of selecting best candidate parents for next improvement cycle, and/or what to release as new cultivars for a given area or large region.

When significant, GEI has an important role in accounting for the phenotypic variation of quantitative traits and can be accommodated in statistical models designed for multi-environmental trials (Cooper *et al.* 1996). Stable genotypic performance is highly desirable in improved cultivars, which is important for food security and industrial uses (Boyer *et al.* 2013). Yield stability is defined as the ability of a genotype to avoid significant fluctuation in yield over a range of environmental conditions (Heinrich *et al.* 1983). However, responsiveness to advances in the agronomic improvement of a production environment is also an important aspect in breeding, which describes the cultivar’s ability to react to the change in the environmental conditions.

The difference between stability and responsiveness can be graphically conceptualized (Figure 1). Cultivar A is stable in environments of low and high agronomic productivity, but that stability comes at the cost of not responding to agronomic improvement. Cultivar B is also stable, and yields higher in both low and high environments than Cultivar A. Cultivar A and B represent an unrealistic situation in a context that involves consideration of genotypic yield response to a range of low-to-high yield environments because varieties do not perform exactly the same in terms of yield when the environment changes significantly. Cultivar C is responsive to improvement in the environmental productivity. With respect to just A and B, their GEI is a noncross-over type (*i.e.*, B is always superior to A in both environments and thus rank *per se* selection will be successful). Cultivar D is higher yielding than either C or E when environmental productivity is low, but is lower yielding than C when productivity is high. In this instance, the greater stability of cultivar D (with less change in yield) *vis-à-vis* the greater responsivity of cultivar C results in a cross-over type of GEI (*i.e.*, an inverted performance rank of C and D), thus making the choice of the best cultivar dependent upon the productivity of the targeted environment. Clearly, when cultivars C and D are compared, cultivar D is favored in environments of low productivity, but cultivar C is favored in environments of high productivity. When cultivars C and E are compared, they both respond to the environmental change but cultivar C is superior to cultivar E in both low and high environments.

**Figure 1:**
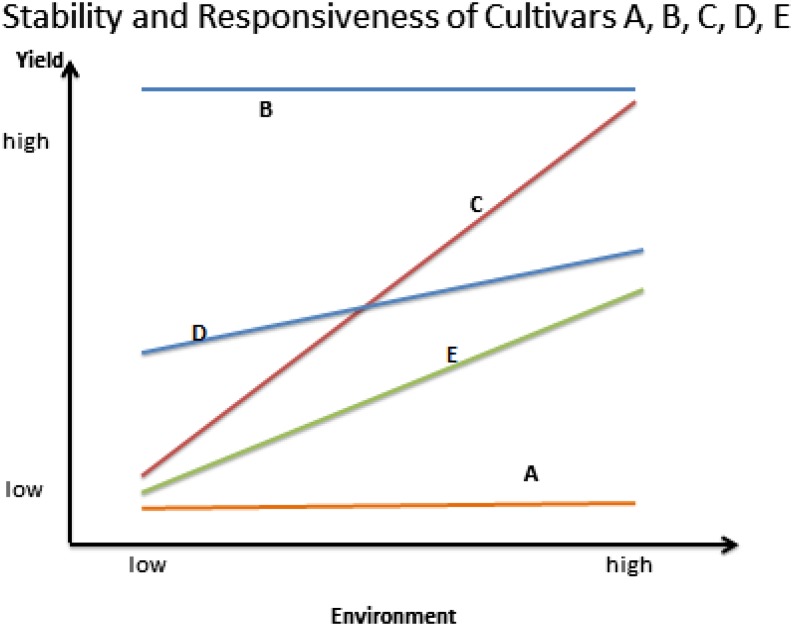
A graphical depiction of the difference between static and dynamic stability; showing the stability and responsiveness of Cultivars A, B, C, D, and E.

Becker and Leon (1988) classified stability as either static or dynamic. Static stability would be typified by a cultivar with a low yield variance (compared to other cultivars) over a range of low to high environmental productivities (*i.e.*, cultivar A in Figure 1). Dynamic stability was characterized as a cultivar whose performance, when regressed across a low to high environmental productivity range, mirrors the overall mean regression performance of all cultivars in the same trial.

Many breeders have used the Finlay and Wilkinson (1963) approach of joint linear regression analysis to assess GEI, by plotting the individual genotypic regression coefficients (*i.e.*, genotypic response to a linear array of environmental productivities) against the genotypic means over all environments to interpret the results (Figure 2). Genotypes with more “stability” have regression coefficients of less than unity, which is consistent with these genotypes performing well in low productivity environments, but also performing poorly in high productivity environments. Genotypes with regression coefficients >1 are more sensitive to environmental changes, thus the environmental conditions have a greater influence on their performance than the genotypes that have regression coefficients closer to zero.

**Figure 2:**
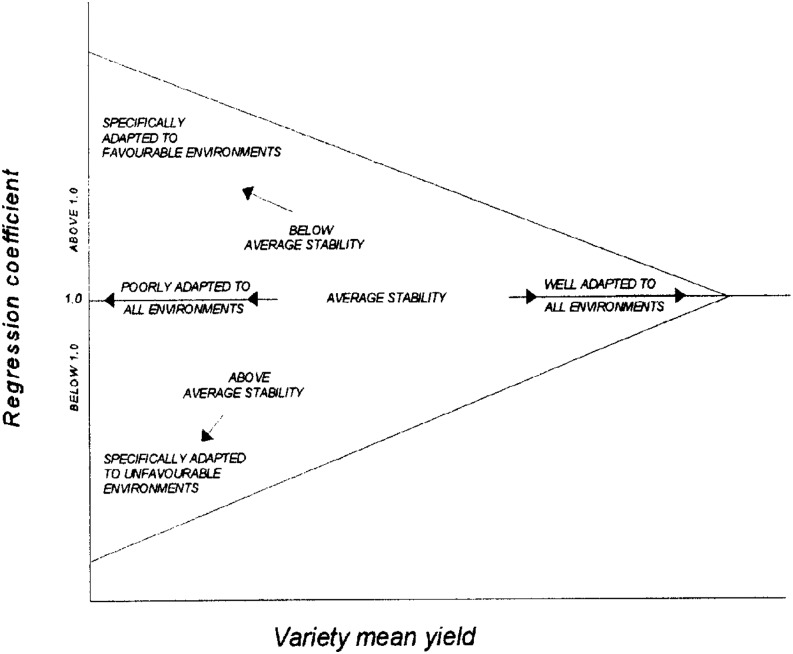
A generic depiction of FW genotype regression coefficients *vs.* genotypic means (from Finlay and Wilkinson 1963).

Easy access to molecular tools in the “omic” era has opened a new horizon to understand and exploit GEIs at the genomic level (Deshmukh *et al.* 2014; Cuevas *et al.* 2017). There is now a growing interest in investigating the genetic basis of GEI at a quantitative trait locus (QTL) level, allowing marker-assisted selection to be brought to the fore in terms of manipulating trait stability or responsiveness to environmental stimuli in breeding programs (Des Marais *et al.* 2013; Malosetti *et al.* 2013).

The behavior of cultivars in distinct environments is of special interest in breeding efforts targeting complex traits, such as grain yield, which are controlled by a large number of alleles, mostly presenting small effects, but which are very responsive to the environment (Des Marais *et al.* 2013; Xavier *et al.* 2016). The development of large experimental multiparental panels, known as next-generation populations (Morrell *et al.* 2012), has made studies of complex traits feasible, as it provides sufficient power and resolution to detect genomic associations for complex traits. Nested Association Mapping (NAM) populations are a special kind of multiparental panel, characterized by multiple biparental crosses that share a common parent.

The NAM structure enabled maize researchers to identify QTL for agriculturally important traits with high statistical power and mapping resolution (Yu *et al.* 2008). Such genetic resource, along with enough phenotypic data, can enable scientists to address questions about the genomic origins of genotype by environment interactions, and, in combination with environmental information yield stability, can be addressed for the genotypes. The first NAM population was developed in maize, and originated from 25 diverse maize lines mated to the inbred line B73, followed by selfing the F1 plants for six generations to generate 200 recombinant inbred lines (RILs) per mating (*i.e.*, family) totaling to 5000 individuals in the maize NAM population, which then was used in multi-environmental scenarios for genomic prediction purposes (Guo *et al.* 2013) to identify GEI.

Another NAM population was created in soybean to take advantage of historic and more recent recombination in the examination of the genetic architecture of complex traits, and the influence of environmental variation with high statistical resolution power. In this study, the main objective was to perform a genome-wide association study (GWAS) involving the soybean NAM population to identify genomic regions that not only influenced grain yield across all environments, but also regions that influenced GEI.

Two standard analysis methods that were previously used to investigate connections between genetics and the environment in soybeans (Zhe *et al.* 2010) were herein adapted to conduct a whole-genome analysis: (a) a regression-based stability index (Malosetti *et al.* 2013), and (b) the principal components-based interaction (Malosetti *et al.* 2013). In the regression-based stability index, the regression coefficient of a linear regression model describes the genotype across different environments, and the deviation from the estimated regression describes the consistency of the genotype. When the regression coefficient is zero, the genotype is considered to be dynamically stable. The principal-component-based interactions can be evaluated via Genotype and Genotype-Environment (GGE) biplot analysis where the GEI can be accessed visually, and the stability of the genotypes and environments can be evaluated.

The stability testing and GEI testing were performed using the soybean NAM population. In this paper, we first describe the pedigree structure of the soybean NAM population, how the phenotypic data were collected in the multiple environments, and how the data were utilized for our analyses. Genome-wide associations were conducted using the Finlay-Wilkinson stability (FW) index (Finlay and Wilkinson 1963) to evaluate the dynamic stability. The GEI analysis was conducted via meta-analysis using genomic markers and phenotypic values from the multiple environments. Six QTL associated with responsiveness of grain yield to environmental factors and one QTL linked to grain yield stability were identified.

## Materials and Methods

### SoyNAM structure

This study was conducted using a soybean nested association mapping population named SoyNAM, which, as of now, is the largest published set of experimental plant population designed for genetic mapping, comprising 5600 recombinant inbred lines (RILs). The SoyNAM was developed by creating 40 biparental matings that involved one common high yielding parent (IA3023) mated to 40 unique parents (Table 1). Note that the 17 NAM parents were high-yielding cultivars or elite breeding lines nominated by eight state breeders, 15 NAM parents had diverse ancestry and originated from R. Nelson’s Agricultural Research Service-United States Department of Agriculture (ARS-USDA) program at University of Illinois, and 8 NAM parents were nominated by J. Specht at University of Nebraska-Lincoln, because of their high-yielding performance in drought conditions. For more descriptive information about the soybean NAM population, including midsummer and mature photos of the NAM parents, the reader can refer to https://www.soybase.org/SoyNAM/.

**Table 1:** The set of parental lines that were used to cross with the common parent, IA3023, and the corresponding SoyNAM family code

Elite Line	Family	Diverse Line	Family	PI Lines	Family
TN05-3027	NAM 2	LG03-2979	NAM24	PI-398881	NAM40
4J105-3-4	NAM 3	LG03-3191	NAM25	PI-427136	NAM41
5M20-2-5-2	NAM 4	LG04-4717	NAM26	PI-437169B	NAM42
CL0J095-4-6	NAM 5	LG05-4292	NAM27	PI-507681B	NAM46
CL0J173-6-8	NAM 6	LG05-4317	NAM28	PI-518751	NAM48
HS6-3976	NAM 8	LG05-4464	NAM29	PI-561370	NAM50
Prohio	NAM 9	LG05-4832	NAM30	PI-404188A	NAM54
LD00-3309	NAM10	LG90-2550	NAM31	PI-574486	NAM64
LD01-5907	NAM11	LG92-1255	NAM32		
LD02-4485	NAM12	LG94-1128	NAM33		
LD02-9050	NAM13	LG94-1906	NAM34		
Magellan	NAM14	LG97-7012	NAM36		
Maverick	NAM15	LG98-1605	NAM37		
S06-13640	NAM17	LG00-3372	NAM38		
NE3001	NAM18	LG04-6000	NAM39		
Skylla	NAM22				
U03-100612	NAM23				

The first two columns of the table represent the name and the family designation of the elite lines, the third and fourth columns are for the genetically diverse lines, and the last two columns are for the plant introduction lines.

**Figure 3:**
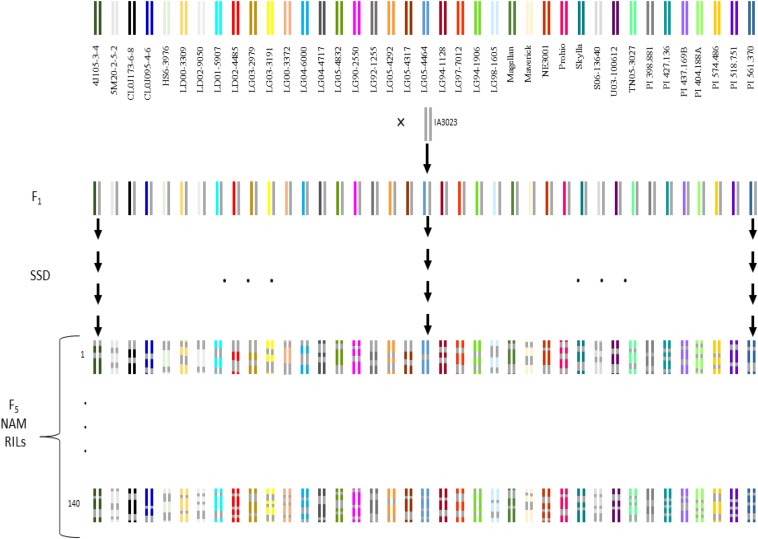
Schematic diagram of the development of the SoyNAM.

The development of the SoyNAM population (Song *et al.* 2017) was similar to the maize NAM population (Figure 3). Each of the 40 biparental matings resulted in 140 F_5_ derived RILs, derived from F2 plants via single-seed descent, and thus totaled to 5600 RILs. Some RILs had to be dropped due to mislabeling, and other generation advance errors, and one family was dropped because of uncertainty relative to the parental ancestry. The final NAM subset utilized for this investigation consisted of 39 families and 5143 RILs.

The RILs were planted at nine locations during the years of 2011, 2012, and 2013, but data were collected on only 18 unique environments (location–year) combinations. Phenotypic data were collected on grain yield (in kg/ha^−1^), days to maturity, plant height (in centimeter), lodging (score 1–5), seed size (mass of 100 seed in grams), plus seed composition (in % in the grain) of protein, oil, and fiber content. For this study, however, the focus was on grain yield adjusted to 13% moisture.

The entire set of RILs were grown at four locations in Nebraska, Iowa, Illinois, and Indiana in 2012 and 2013, but the 2013 Nebraska trial was unfortunately lost due to a hail storm. Only partial RIL sets were grown at other locations in one or more of the 3 yr. The number of RILs grown in the 18 environments was somewhat variable due to some missing plots (Table 2). The locations in Ohio were divided into two regions; the South Charleston area and the Wooster area. A considerable number of check cultivars (*i.e.*, parents, high yield checks, and maturity checks) were grown in each trial environment to assess field variation (Table 3).

**Table 2:** Number of SoyNAM RILs with nonmissing plot data in each environment

	Iowa	Illinois	Indiana	Kansas	Michigan	Missouri	Nebraska	Ohio1	Ohio2
2011	—	2500	—	—	—	—	2500	—	—
2012	5111	5138	5041	3158	816	819	5127	1606	1626
2013	5100	5137	5136	3230	—	804	—	1619	1571

**Table 3:** Number of check cultivars in each environment

	Iowa	Illinois	Indiana	Kansas	Michigan	Missouri	Nebraska	Ohio1	Ohio2
2011	—	419	—	—	—	—	419	—	—
2012	825	825	825	525	125	125	825	250	253
2013	825	825	825	510	—	137	—	253	262

The experimental units were two-row plots (2.9 × 0.76 m), seeded at a density of *ca*. 36 plants/m^2^, arranged in a modified augmented design (Lin and Poushinsky 1983) without replication within each test environment. Yields were restricted by drought conditions during 2012 (Rippey 2015) although plots were always irrigated at Nebraska and Kansas.

The average grain yield of the RILs in the 18 environments ranged from a low of 2364 kg ha^−1^ in 2012 in Michigan to respective highs of 5057 and 5050 kg ha^−1^ in 2011 in Nebraska and 2013 in Indiana (Table 4).

**Table 4:** Average grain yield (kg ha^−1^) of SoyNAM RILs observed in each environment

	Iowa	Illinois	Indiana	Kansas	Michigan	Missouri	Nebraska	Ohio1	Ohio2
2011	—	2780	—	—	—	—	5057	—	—
2012	2776	3386	4231	3871	2364	3414	4728	3394	2823
2013	2871	3115	5050	2747	—	4091	—	3629	4420

**Figure 4:**
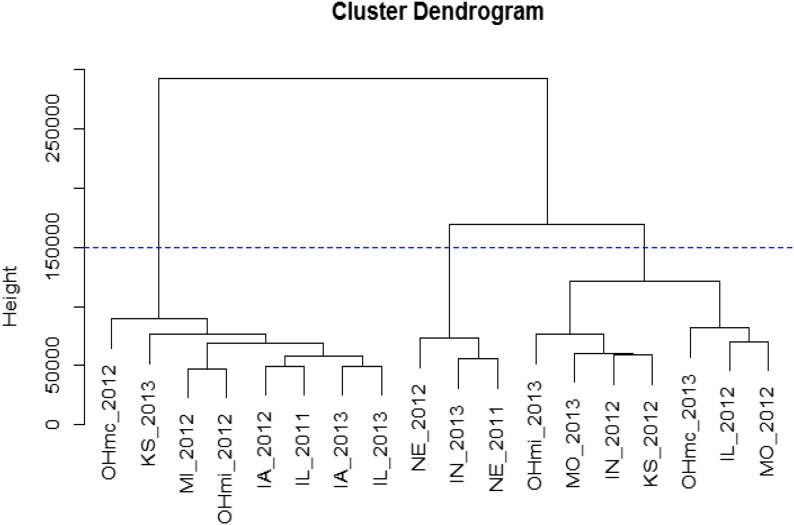
Hierarchical clustering of SoyNAM environments based on grain yield data based Ward D method and Euclidean distance.

The yield data collected from the 18 environments were used to identify clusters of similarity among the environments (Figure 4). The cluster dendogram was created using a hierarchical clustering algorithm *hclust* implemented in R (R Core Team 2017) with the Ward-D agglomeration method using the Euclidean distance. The OHmc location represents the South Charleston, Ohio area, which was obtained by L.M., and the OHmi location represents the Wooster, Ohio area which was the responsibility of R.M. The eight site-year trials in the leftmost cluster were the distinctively lower yielding (*i.e.*, <3400 kg ha^−1^) than the three rightmost clusters. Of the latter, the central cluster with the three site-year trials of NE 2011 and 2012 and Indiana 2013 was the highest yielding (*i.e.*, >4700 kg ha^−1^), and was distinctly different from the other two clusters of four and three site trials (*i.e.*, yield range of 3386–4420 kg ha^−1^).

### Genotyping

Parental lines were sequenced to derive the SNP allele calls. A total of 5303 SNP loci were selected with the criterion of maximizing the number of families segregating for those loci. The SNPs were used to build the SoyNAM 6K BreadChip SNP array using the Illumina Infinium HD Assay platform (Illumina, Inc.) (Song *et al.* 2017). Among those SNPs, a subset of 4312 markers were selected by the SoyNAM group as quality-assured based on proportion of missing loci and correct segregation patterns. Both raw and quality assured genotypes are available in the R package SoyNAM. The missing genotypes were imputed using a random forest algorithm (Stekhoven and Bühlmann 2012).

The statistical model designed for association in multiple populations (Wei and Xu 2016) requires the markers to represent the parental sources, so we proceeded as follows: SNPs were recoded to express the allele of origin, where 0 represents homozygous for the founder parent, 1 as heterozygous, and 2 homozygous for the common parent IA3023. Using the allele and family information, the R package NAM (Xavier *et al.* 2015) recoded each locus into an incidence vector indicating the parental origin of the alleles, in other words, as the interaction marker-by-family, hence allowing markers to present different effects across families.

### Association analysis

Genome-wide association analyses were based on the multiparental model proposed by Xavier *et al.* (2015) and implemented for NAM populations. A detailed theoretical description is presented by Wei and Xu (2016), who extended the methodology to multiparent advanced generation intercross-population (MAGIC) populations. The following mixed linear model describes how the association analyses were performed:

y=Xβ+Zα+ψ+e (1)

where **y** is the vector of phenotypes, **X** is the design matrix of fixed effects allocating the intercept and block effect estimated from checks, **β** is the fixed effect coefficients, **Z** is the incidence matrix of the marker data, **α** is the vector of regression coefficients associated with the haplotypes, **ψ** corresponds to the polygenic coefficients, and **ε** is the vector of residuals. The model assumes that **α** ∼ N(0, $I{\text{σ}}_{\text{α}}^{2}$), ψ ∼ N(0, $K{\text{σ}}_{\text{ψ}}^{2}$) and ε ∼ N(0, $I{\text{σ}}_{\varepsilon }^{2}$), where ${\text{σ}}_{\text{α}}^{2}$ is the genetic variance associated with the haplotypes, ${\text{σ}}_{\text{ψ}}^{2}$ is the genetic effect associated with the polygenic effects. The population structure is captured by the polygenic term with covariance **K**, which corresponds to the genomic relationship matrix that represents the genetic similarities among individuals through a linear additive kernel ($K=\text{α}MM^{\prime },$ Xu 2016).

Statistical significance of single markers was evaluated through the likelihood ratio test (LRT) by comparing the log-likelihood of the model that includes the marker effect Zα (${\text{L}}_{1}$) with the log-likelihood of the model that does not (${\text{L}}_{0}$), *i.e.*, the reduced model. ${\text{L}}_{0}$ represents the null hypothesis, and L_1_ represents the alternative hypothesis. The LRT (McCulloch and Searle 2001) for comparing the full model (L_1_) to the reduced model (L_0_) can be written as

$$\text{LRT}=-2({\text{L}}_{1}-{\text{L}}_{0}).$$ (2)

In the random effects model, the LRT follows a mixture of chi-square and binomial distributions (Xavier *et al.* 2015), with p-values computed using a chi-square distribution with 0.5 degrees of freedom. Multiple test correction was performed via a Bonferroni threshold (α=0.05) to define which markers were significantly associated with the FW index. The Bonferroni threshold for 4312 SNPs is a −log (p-value) value of ∼5.

### GEI testing

Association analyses for grain yield were conducted for individual environments one at a time using the described model in (1). Meta-analysis, which combines data from multiple sources for analysis was utilized to infer markers that presented significant GEI from the estimated allele effects when using grain yield as response variable. Meta-analysis was performed with the following model:

α=Wδ+e (3)

where **α** is the vector of allele effects estimated from the association analysis for individual environments, **W** is the incidence matrix of parental source (where each column of W refers to a parent, each element of α is the marker effect of a family in a given environment), δ is the vector of regression coefficients representing the true marker effects, and e is the vector of residuals. The environmental term is not added to the GGE model (Yan *et al.* 2007). A GGE model is a parametric, multivariate technique used to predict the adaptation and stability of a cultivar (Mortazavian *et al.* 2014), based on environment-centered principal components (Gauch 2006). Environment-centered principal components refer to the technique in which singular value decomposition (SVD) is applied to the residual matrix resulting from the environmental mean estimation.

The interaction term (**γ**) in model (4) is defined as a matrix with dimensions corresponding to the number of NAM parents by the number of environments whose entries are comprised with the first component from the single value decomposition of the residuals. The model is

α=Wδ+γ+ϵ (4)

where W and δ are as defined above, **γ** represents the interaction term, and ϵ is the new vector of residuals, without the GE interactions captured by γ. The ${\text{γ}}_{ij}$ multiplicative interaction of the ith family at the jth environment can be described as the product of the first set of eigenvectors (**U** and **V**) and the first eigenvalue (**D**) from the residual single value decomposition as follows:

$${\text{α}}_{\mathrm{ij}}-\sum {\text{w}}_{j}{\text{δ}}_{j}={\text{e}}_{ij}={\text{γ}}_{ij}+\epsilon_{ij}=u_{\text{i}}{\text{d}}_{\text{ii}}v_{\text{j}}+\epsilon_{ij}\;\;(i=\text{fam},j=\text{env})$$ (5)

Models (3) and (4) were fitted via weighted least squares, where the allele effects were weighted according to the number of individuals observed in each combinations of NAM family and environment. The statistical test is a likelihood ratio, as presented in (2). The model in (3) is assumed to represent the null hypothesis, whereas the model presented in (4) represent the alternative hypothesis. The LRT was assumed to have a chi-square distribution, where the number of degrees of freedom is a function of the number of environments in which the allele was observed and the number of parents segregating for that allele, which represents the genetic component (Gauch 1988). An empirical threshold of 50 [−log(p-value)] was chosen to define significant associations.

### Data availability

All data are publically available in the R package SoyNAM. Access with the following command: data (soybase, package=“SoyNAM”). Data formatted for the analysis of the NAM package is available with the following command: SoyNAM::ENV().

## Results

**Figure 5:**
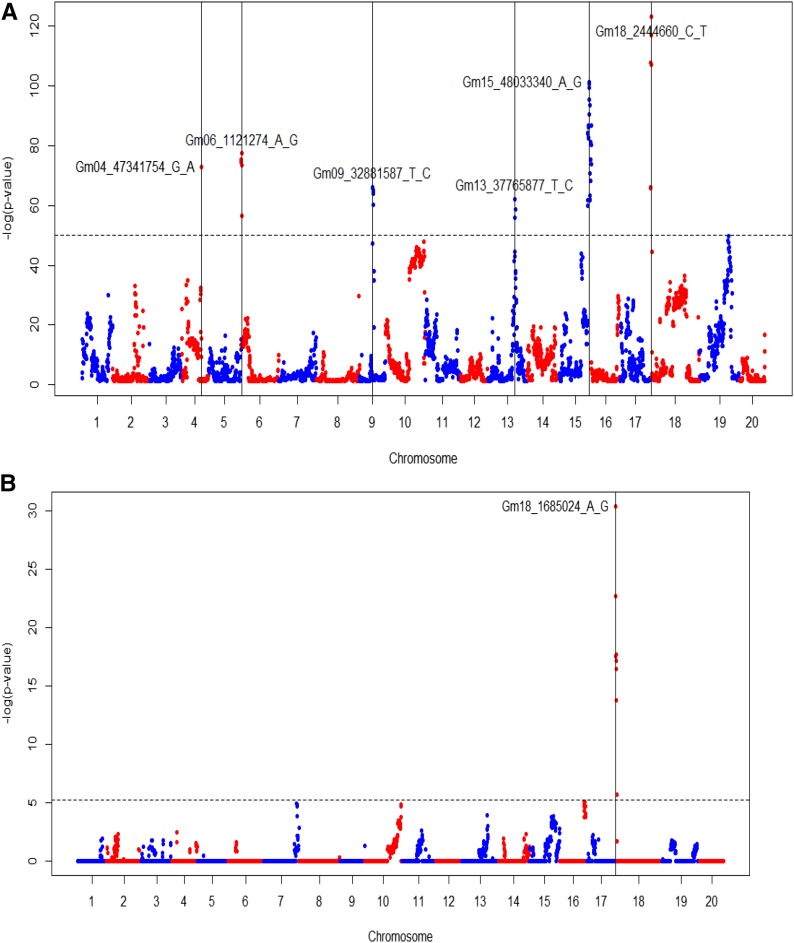
Genome-wide associations to environmental responsiveness. (A) Meta-analysis of the additive main effects and multiplicative interactions. (B) Associations with yield stability using the FW index with Bonferroni significance threshold.

A meta-analysis of GEI identified six statistically significant peaks on chromosomes 4, 6, 9, 13, 15, and 18 (Figure 5A). Among these peaks, the QTL on chromosome 18 also appears to be significantly associated with grain yield stability, as measured with the FW index (Figure 5B).

The most significant SNPs from each QTL, and the annotations of the nearest gene from SNP physical position, are presented in Table 5. The annotation does not necessarily correspond to candidate gene due to the limitation of dataset, including the large linkage disequilibrium block (Hyten *et al.* 2006, 2007) and genotyping coverage in the SoyNAM population (Song *et al.* 2017). However, previous reports were traced in the soybean database “soybase” (Grant *et al.* 2010) supporting the involvement of these genes.

**Table 5:** Summary statistics of the significant QTL positions associated with GEI and stability in the SoyNAM data, and annotation from JBrowse Phytozome (Goodstein *et al.* 2012)

Marker	Trait	Adj R^2^	PC Load	-log(p)	Adj R^2^	Nearest Gene	Annotation*^a^*
Gm04_47341754_G_A	GEI		0.23	72.83		Glyma.04g200700	rRNA 2-O-Methyltransferase Fibrillarin
Gm06_1121274_A_G	GEI	—	0.30	77.40	—	Glyma.06g014900	Protein cup-shaped cotyledon
Gm09_32881587_T_C	GEI	—	0.22	66.19	—	Glyma.09g132200	Beta-carotene 3-Hydroxylase
Gm13_37765877_T_C	GEI	—	0.20	62.11	—	Glyma.13g276100	VQ motif
Gm15_48033340_A_G	GEI	—	0.24	101.38	—	Glyma.15g252200	Glutathione S-Transferase
						Glyma.15g252100	Gibberellin 2-Beta-Dioxygenase
Gm18_1685024_A_G	GEI	—	0.35	123.12	—	Glyma.18g127100	Protein NRT1
Gm18_2444660_C_T	Stability	0.0126	—	30.35	0.0126	Glyma.18g145700	UDP-glucose 4-epimerase

a Annotation from JBrowse Phytozome (Goodstein *et al.* 2012).

The chromosome 18 QTL associated with stability explained 1% of the variance of the FW index (Table 5), and its effect on yield and stability is presented in the Appendix. Regarding the QTL peaks of GEI (Table 5), from 20 to 35% of the variation attributed to interactions between family and environment was captured in the first principal component.

### Genes surrounding the peaks

The genes potentially linked to the regions associated with GEI, or yield stability, have been reported to be involved in environmental stress tolerance or in biosynthetic pathways that may underlie overtly measurable secondary traits such as yield stability and GEI. Details of each gene are presented below.

The rRNA 2-O-Methyltransferase Fibrillarin gene Glyma.04g200700, which corresponds to the SNP peak on chromosome 4 (Table 5), has been reported to be associated with responsiveness to flooding stress in soybeans (Komatsu *et al.* 2012; Won Oh *et al.* 2014). These regions also coincides with the flood stress resistance QTL previously reported by Githiri *et al.* (2006).

The protein giving rise to a cup-shaped cotyledon trait is an enzyme transcribed by the gene Glyma.06g014900, which has a position that corresponds to the peak on chromosome 6 (Table 5). This gene has been reported to be related to seed protein storage in soybeans (Han *et al.* 2009), and it was previously reported in the same region through QTL mapping (Ma *et al.* 2016). In another QTL study, Hwang *et al.* (2013) reported this region related to ureide content in soybeans, used as an indirect measure of drought resistance.

Beta-carotene 3-hydroxylase is the enzyme transcribed from the gene Glyma.09g132200, which corresponds to the peak on chromosome 9 (Table 5). This protein is associated with enhanced stress tolerance in *Arabidopsis* (Davison *et al.* 2002), by preventing photo-oxidative damage caused by excessive light. Genetic signals for resistance to ultraviolet β radiation were previous report in the soybeans chromosome 9 by Shim *et al.* (2015). β-Carotene hydroxylases have also been associated with nodulation in soybeans (Kim *et al.* 2013).

Glyma.13g276100, which corresponds to the SNP peak on chromosome 13 (Table 5), produces VQ motif, which has been reported to play an important role in abiotic stress, plant development, and nitrogen metabolism in soybeans (Wang *et al.* 2014).

The SNP Gm15_48033340_A_G has a genomic position close to two genes: Glyma.15g252200 and Glyma.15g252100 (Table 5). Glyma.15g252200 is transcribed into Glutathione S-Transferase (GST), which plays an important role in soybean development (McGonigle *et al.* 2000), and is associated with salt and drought tolerance in soybeans (Ji *et al.* 2010). GST is also related to mycorrhiza symbiosis (Hogekamp and Küster 2013), which may be involved in water and nutrient absorption. The second gene, Glyma.15g252100, is transcribed into Gibberellin 2-Beta-Dioxygenase, an enzyme involved in soybean nodulation (Zhu *et al.* 2013) and nodule activity (Méndez *et al.* 2014). The enzyme is also known to interfere with plant growth, root gravity, and salt stress tolerance (Shan *et al.* 2014).

The SNP Gm15_48033340_A_G also coincide with two QTL previously reported to be associated with resistance to root-knot nematode (Tamulonis *et al.* 1997) and soybean cyst nematode (Yue *et al.* 2001).

Two peaks were found on chromosome 18 (Table 5). Glyma.18g127100 (*i.e.*, Nitrogen Transporter – 1, NRT1), which was the gene identified by SNP signal for stability, produces NRT1, which is responsible for the transport of nitrogen metabolites, and it is believed to play a role in the soybean nitrogen uptake (Yokoyama *et al.* 2001).

Several studies in *Arabidopsis*, maize and soybean have established correlation between grain yield with nitrogen remobilization and nitrogen storage capacity (Fan *et al.* 2009). In addition, soil nitrogen changes have been shown to produce improvements in grain yield and Nitrogen Use Efficiency (NUE) (Ma *et al.* 1999).

Nitrate Transporters such as NRT1 play key roles in nitrogen remobilization, with several members of NRT1 family performing specific roles in nitrogen uptake, efflux, and tissue-to-tissue nitrogen transport (Parker and Newstead 2014; Tsay *et al.* 1993; Lin *et al.* 2008). Given its plausible indirect effect on yield, the role of NRT1 in yield stability is not clear, as we do not have data on soil nitrogen level and other confounding factors in the different environments.

Glyma.18g145700, which is the nearest gene from the QTL identified on chromosome 18 for GEI, produces UDP-glucose 4-epimerase (BrUGE). BrUGE appears to be involved in the cell wall carbohydrate partitioning under nitrogen stress in rice (Guevara *et al.* 2014) and *Arabidopsis* (Barber *et al.* 2006). Epimerases have been found to be N-responsive, showing expression levels in N-dependent manner (Guevara *et al.* 2014). This further hints at the correlation of N-metabolism with yield stability.

## Discussion

In yield trials, plant breeders evaluate genotypes in multiple environments to identify the superior ones, but the presence of GEI complicates the attainment of this objective. Ideally, genotypes that perform well across a broad range of environments are desired, though genotypes with specific superior yield responses in the lowest *vs.* highest ends of the range of environments may require selection of genotypes with specific adaptation (Figure 2). Still, it is important to partition environmental and genetic variability to determine how GEI impacts these variances, and how stable or responsive the selected cultivars are to environmental stimuli.

### Abiotic stress

Soybean cultivars that display genetic tolerance to different stresses are more likely to endure suboptimal environmental conditions (Des Marais *et al.* 2013). The nearest genes colocalized with the QTL peaks were reported in literature to be related to the physiological response of soybeans and other species to various types of stresses. In summary, these genes were reported to play a role in soybean flood, drought and salt tolerance (Chr 4 and 15), tolerance to photo-oxidative stress (Chr 9), plant development, nodulation, and nitrogen metabolism under stress (Chr 9, 13, and 15), and water and nutrient uptake (Chr 15 and 18).

Tolerance to different stresses can frequently be inter-related. For instance, soybean nitrogen fixation is favored under optimal environmental conditions, such that minor abiotic stresses can disrupt the process (Salvagiotti *et al.* 2008). Water-related stresses inhibit nodulation by restricting photosynthate supply to nodules (Gil-Quintana *et al.* 2013). However, genotypes with enhanced enzymatic degradation of ureide are drought tolerant by being able to perform nitrogen fixation under stress (Purcell *et al.* 2000; Sinclair *et al.* 2007; Ray *et al.* 2015).

### Associations in multiple environments

Genome-wide analysis including interactions between markers and environment has been scarce in plant due to the lack of statistical power (El-Soda *et al.* 2014). Attempts to identify GEI through genome-wide scans have been performed in barley (Malosetti *et al.* 2004) and maize (Boer *et al.* 2007) in a multi-step approach based on regressing the QTL effects on environmental factors to detect interactions. These studies also found few regions significant for GEI, which indicates an oligogenic architecture for interactions where only some genomic regions were responsive to environmental stimuli.

### Quantitative nature of secondary traits

Stress-related genes are important when the crop is exposed to harsh environments. Genomic regions that contain these genes are unlikely to show significant association to yield when the phenotypic data were collected from optimal environmental conditions, because the phenotypic variation attributed to these genes is hidden under stress-free seasons (Schlichting and Smith 2002). However, the inverse may also be true, in that productivity-improving genes may not be readily detectable in low productivity environments.

In this context, GEI and stability are secondary traits used to investigate variation of a primary trait of interest—grain yield. Figure 5 indicates that a number of genomic regions were responsive to environmental stimuli. The environment-dependent expression of these regions modulates the phenotype under unfavorable conditions (Smith 1990), resulting in the stress-related genes to play a major role to the stability of grain yield. On another hand, De Bruin and Pedersen (2009) reported that cultivars resistant to soybean cyst nematode were able to maintain good performance standards in low yielding environments, being more stable than their susceptible counterparts.

### GEI, stability, and breeding

Breeding soybeans for high yield can be translated into the development of cultivar able to not only thrive under adverse farming conditions, but also have the ability to be responsive to optimal management practices (Board and Kahlon 2011; Ramachandra *et al.* 2015). In other words, breeders desire to increase the genetic yield baseline along with yield potential. For instance, modern soybean cultivars outperform older cultivars while presenting stability index >1 (Rincker *et al.* 2014). The challenge is breeding for both higher yield performance and stability often presents negative genetic correlation (Rosielle and Hamblin 1981).

In order to achieve an ideotype that is both high yielding and stress tolerant the target environments, it is important to be aware of biotic and abiotic stresses in that region, and which “omic” tools provide the necessary information to address the issue (Mir *et al.* 2012). Those may include phenomic sensors, genome-wide selection, or the introgression of QTL that confer stability or yield advantage to the specific target environment (van Eeuwijk *et al.* 2010). In addition, if the genetic progress toward regional ideotypes is slow through conventional methods, or if the exiting genetic resources are scarce, the knowledge of the genes involved in GEI and stability can still provide target regions for genetic engineering (Manavalan *et al.* 2009).

Interactions between the environment and genetics occur in the genomic regions that respond to the environmental stimuli (Smith 1990). We show here that genomic regions exist with strong GEI signaling (Figure 5). The knowledge of the genes that are possibly involved, their functionality and location allow us to strategize the best ways of making good use of these regions.

### Role of NAM

The identification of QTL that modulate grain yield under complex interactions between genomic regions and environmental stimuli can be improved by using multi-parental families with large progeny numbers, which are generated in a NAM project. Aside from the greater statistical power associated with the large number of individuals, the multi-parental model (Wei and Xu 2016) allows for the estimation of SNP effects from each parent–environment combination. Such information is particularly valuable by informing which NAM parent provided the most favorable haplotype in each environment or set of environments, similar to *which-who-where* analysis (Yan 2016). Such variability of the allele effects across families and environments is particularly important for QTL introgression.

**Figure 6:**
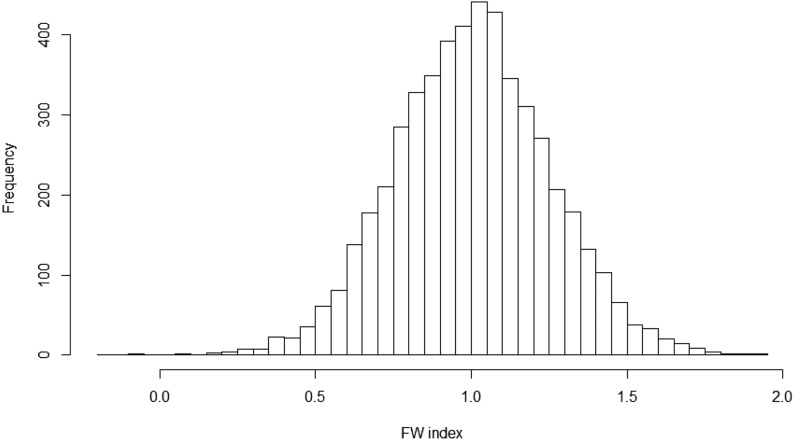
Histogram of FW index of grain yield stability.

Multi-parental association mapping informs which parental lines display the most optimal haplotype that should be used as QTL donors (Xavier *et al.* 2015; Wei and Xu 2016). An example is shown in Table 6 and Figure 6 by demonstrating the FW index for 19 different NAM parents. Estimated allele effect of SNP Gm18_1685024_A_G on grain yield is shown in the second column of Table 6 for the 19 NAM parents, and the FW index is shown in the third column of Table 6. The index is a deviation from a regression coefficient $b_{FW}=1$ showing the contribution of GEI. Since the FW index is low for all of the NAM parents it shows that the effect of this QTL is influenced by the environment, thus showing GEI. This statistical procedure for associations acknowledges the possibility that the same allele may have different effects in different families and environments.

**Table 6:** Stability peak on chromosome 18 for the NAM parents segregating for the SNP

Allele Donor	Yield (kg ha^–1^)	FW Index
Intercept	3663.52	0.899
NAM3	58.44	−0.253
NAM4	−6.96	0.236
NAM5	20.91	−0.262
NAM10	44.05	−0.306
NAM11	21.54	−0.042
NAM12	8.82	−0.172
NAM13	13.69	−0.035
NAM14	−23.89	0.057
NAM15	2.94	−0.289
NAM18	−5.21	0.240
NAM27	72.91	−0.267
NAM28	−15.95	0.233
NAM33	−42.27	0.045
NAM36	−11.05	0.185
NAM39	−28.09	0.133
NAM48	−27.23	0.132
NAM50	−42.17	0.076
NAM64	−44.43	0.064

The allele effect of Gm18_1685024_A_G on grain yield and upon stability expressed by the FW index.

The distribution of the FW index of grain yield stability is normal with the center ∼1 (shown in Figure 6) demonstrating that most of the positions have no GEI effect on the grain yield. However, the tails of the distribution confirm the existence of GEI in the 19 NAM parents for grain yield stability.

The interaction between family and QTL can be observed when, for instance, the linkage phase between the SNP marker and the QTL is in coupling in some families and repulsion in others (Lynch and Walsh 1998). Another possibility is the interaction between the QTL and the genetic background (Liao *et al.* 2001), which may have implications in the direction and magnitude of allele effects in different families. These scenarios can be allied to an additional order of interactions: between QTL and environment (Wang *et al.* 1999; Xing *et al.* 2002; van Eeuwijk *et al.* 2010).

On another hand, if the panel has a sufficiently dense coverage, noninteractive association techniques can be applied in NAM populations to search for “universal” QTL (Palomeque *et al.* 2010) and causative quantitative trait nucleotides, thus alleles with stable performance across families or environments. Tian *et al.* (2011) pinpointed a few genes associated with leaf architecture in maize using a NAM population with high-density genotyping. For the SoyNAM, such approach can be enabled by the imputation or projection of the current SNP panel of 4323 SNPs into a higher-density platform (Howie *et al.* 2011).

### Conclusion

Stability analysis can aid plant breeders in the selection procedure, and give cultivar recommendations. Identifying the chromosome regions that influence stability can further enhance the selection process. In this study we evaluated the multi-parental population referred to as the SoyNAM population, and conducted genome-wide stability analysis with the FW index as the phenotype, and genome-wide meta-analysis based on the AMMI analysis estimates of the GEI component. We identified six chromosomal regions that were responsible for the GEI, and we found that one of these chromosomal regions was also associated with yield stability.

The success of stability analysis, and the proportion of the phenotypic variability explained by GEI, can be influenced by genotypes and the population structure. In this study, we evaluated a family structure in soybean consisting one common parent crossed with 39 different parents. Stability analysis was performed using a nested family structure in multiple environments, which led to the identification of chromosome segments that are associated with yield stability and GEI.

We envision future direction of this research as the investigation of grain yield stability conditional to different environmental representation: genome-wide prediction studies of yield performance is needed along with enviro-typing (Xu 2016), targeting the soybean genomic assisted breeding research through transdisciplinary investigations involving genetics and precision agriculture. The representation of management and environmental parameters in continuous scale (Lee *et al.* 2016) could represent a potential emerging area for genomic studies of GEI and stability in soybeans.
